# Hallucination Rate of Peer-Reviewed Citations Generated by Large Language Models in Neurocritical Care

**DOI:** 10.1097/CCE.0000000000001474

**Published:** 2026-08-25

**Authors:** Ali Seifi, Ali Seyfi

**Affiliations:** 1 Department of Neurosurgery, University of Texas Health San Antonio, San Antonio, TX.; 2 Machine Learning and NLP Lab, Department of Computer Science, George Washington University, Washington, DC.

**Keywords:** artificial intelligence, citation hallucination, fabricated references, large language models, neurocritical care

## Abstract

**IMPORTANCE:**

Large language models (LLMs) are increasingly used for scientific literature retrieval, yet their citation accuracy in specialized clinical domains remains poorly characterized. In neurocritical care (NCC), fabricated or inaccurate citations may be difficult to detect without deliberate verification.

**OBJECTIVES:**

To evaluate hallucination and fabrication rates of peer-reviewed citations generated by three LLMs across core NCC topics, under constrained zero-shot, memory-only conditions.

**DESIGN, SETTING, AND PARTICIPANTS:**

In this cross-sectional, blinded technology performance evaluation, Generative Pretrained Transformer (GPT)-5.3, DeepSeek-V3, and Grok-4 were queried on March 10, 2026, under identical zero-shot, retrieval-disabled web-interface conditions. Ten NCC topics were submitted to each model, and each model generated 10 references per topic, yielding 300 references.

**MAIN OUTCOMES AND MEASURES:**

Two NCC experts, blinded to model identity, independently verified each reference against PubMed, DOI, Google Scholar, and CrossRef and scored accuracy using a Hallucination Scale (0–3). The primary outcome was any hallucination, defined as any citation inaccuracy. The secondary outcome was fabrication, defined as a nonexisting complete bibliographic entity.

**RESULTS:**

Inter-rater agreement was excellent (κ = 0.91; 95% CI, 0.86–0.96). Overall, 165 of 300 references (55.0%) contained a citation inaccuracy, and 85 of 300 (28.3%) were completely fabricated. DeepSeek-V3 had the lowest hallucination rate (23%; fabrication 8%), followed by GPT-5.3 (69%; fabrication 27%) and Grok-4 (73%; fabrication 50%). Compared with DeepSeek-V3, Grok-4 was 3.17 times more likely to hallucinate (95% CI, 2.03–4.96; *p* < 0.001), and GPT-5.3 was 3.00 times more likely to hallucinate (95% CI, 1.94–4.63; *p* < 0.001). Topic-level findings were exploratory and should be interpreted cautiously.

**CONCLUSIONS AND RELEVANCE:**

Under standardized zero-shot, retrieval-disabled web-interface conditions, LLMs generated substantial numbers of inaccurate and fabricated NCC citations. Because fabricated references can appear complete and credible, artificial intelligence-generated citations should be verified across reliable databases before use in clinical, educational, or scholarly work.

**KEY POINTS**

**Question:** Do large language models generate accurate peer-reviewed citations in neurocritical care (NCC) under standardized zero-shot, retrieval-disabled conditions?

**Findings:** In this cross-sectional evaluation of 300 model-generated references across 10 NCC topics, 55.0% contained citation inaccuracies, and 28.3% were completely fabricated. DeepSeek-V3 had the lowest hallucination and fabrication rates, followed by Generative Pretrained Transformer-5.3 and Grok-4. Topic-level variation was observed, but it should be interpreted with caution.

**Meaning:** Under zero-shot conditions, large language model-generated NCC citations remain unreliable without verification. artificial intelligence-generated references should be checked across reliable databases before clinical, educational, or scholarly use.

The rapid advancement of artificial intelligence (AI), particularly the large language models (LLMs) is evolving as a new era for scientific writing, with tools such as Chat Generative Pretrained Transformer (ChatGPT), DeepSeek, and Grok demonstrating remarkable capabilities in generating coherent, human-like text across diverse domains, including medicine (1, 2). These models are increasingly being used by clinicians and trainees to assist with literature synthesis, hypothesis generation, and article preparation, offering the potential to accelerate research productivity and enhance scientific communication (3). However, alongside these promising applications, a critical concern has emerged: a new form of “hallucination,” distinct from the perceptual medical condition familiar to clinicians, wherein AI models generate plausible-sounding but factually incorrect content, including fabricated references and inaccurate scientific claims (4, 5). AI hallucination arises because LLMs generate responses based on learned patterns from training data rather than verified factual knowledge, predicting the most probable next word sequence without internal mechanisms for truth validation (6). This issue is particularly consequential in high-stakes fields such as critical care, where erroneous information could directly affect patient safety and clinical decision-making (7). Recent evaluations demonstrate that hallucinations, including reference fabrication, remain pervasive across multiple LLMs, even in expert-reviewed settings (4, 8, 9).

In critical care, where decisions are time-sensitive and high-risk, the application of LLMs is rapidly expanding across clinical decision support, documentation, and education, including their use in generating scientific writing and research backgrounds (10, 11). Nevertheless, hallucinations and concerns about reliability pose significant barriers to safe implementation, and risks of uncritical adoption become increasingly apparent (11). Studies have documented hallucination rates exceeding 50% in certain medical writing tasks, with models confidently producing fabricated citations and misleading clinical recommendations (8, 9). Neurocritical care (NCC) amplifies these risks further. Decisions regarding intracranial pressure management, refractory status epilepticus, and neuroprognostication after cardiac arrest depend on a precise, evidence-based foundation; erroneous citations incorporated into clinical reasoning, educational materials, or guideline submissions in this specialty may create downstream patient-safety risk if accepted without verification (7). Despite this risk, no prior study has systematically evaluated LLM citation hallucination rates in any critical care subspecialty.

We therefore evaluated three LLMs (GPT-5.3, DeepSeek-V3, and Grok-4) across 10 core NCC topics, comparing model- and topic-level hallucination rates. Using a standardized, retrieval-disabled design to evaluate citation generation under constrained zero-shot, memory-only conditions, we hypothesized that hallucination rates would vary significantly by both model and clinical domain.

## METHODS

**Figure 1.**
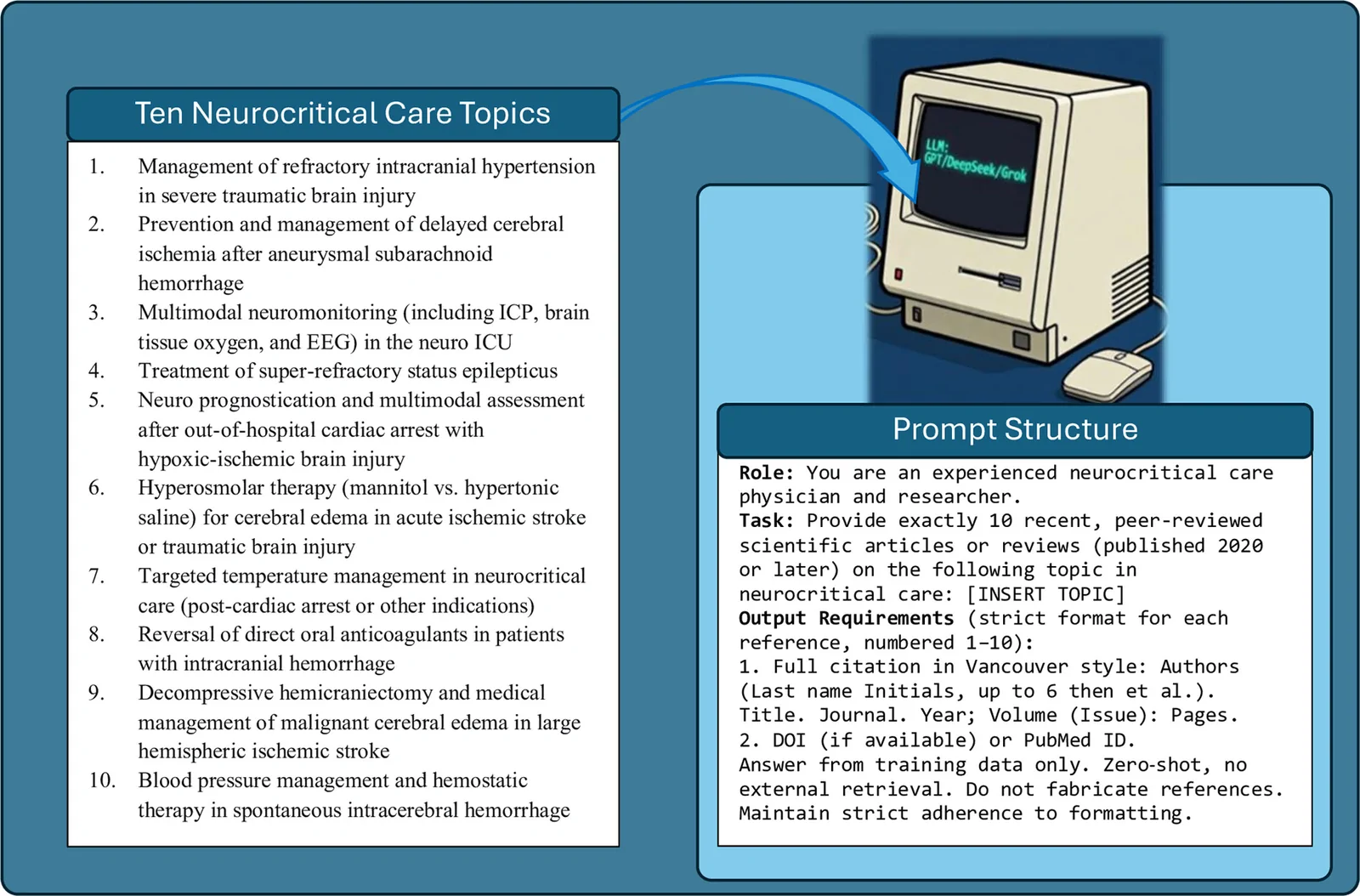
Study design and standardized prompt workflow. Ten neurocritical care topics were each submitted to three large language models—Generative Pretrained Transformer (GPT)-5.3 (ChatGPT; OpenAI), DeepSeek-V3 (DeepSeek AI), and Grok-4 (xAI)—using an identical zero-shot prompt instructing the generation of 10 recent peer-reviewed references from training data only, yielding 300 reference outputs (10 topics × 3 models × 10 references). Each reference was independently verified by two blinded, domain-expert reviewers against PubMed, DOI resolvers, Google Scholar, and CrossRef, and scored using a modified Reference Hallucination Scale (0–3): score 0 = fully accurate, 1 = minor error/citation inaccuracy (typo in author, title, volume, or page), 2 = major error/citation inaccuracy (wrong title, author, journal, or year), 3 = completely fabricated. The 10 neurocritical care topics evaluated were: 1) refractory intracranial pressure, 2) aneurysmal subarachnoid hemorrhage and delayed cerebral ischemia, 3) multimodal neuromonitoring, 4) super-refractory status epilepticus, 5) neuroprognostication after cardiac arrest, 6) hyperosmolar therapy, 7) targeted temperature management, 8) direct oral anticoagulants reversal in intracerebral hemorrhage, 9) malignant middle cerebral artery stroke, and 10) blood pressure management in spontaneous intracerebral hemorrhage.

This cross-sectional, comparative, observer-rated study evaluated the accuracy of AI-generated scientific references across three LLMs in NCC, under constrained zero-shot, memory-only conditions. Ten clinically relevant NCC topics were selected by a domain expert to represent core practice domains (**Fig. 1**). Three widely used models were evaluated: ChatGPT (GPT-5.3; OpenAI, San Francisco, CA), DeepSeek (DeepSeek-V3; DeepSeek AI, Hangzhou, China), and Grok (Grok-4; xAI), hereafter referred to as GPT, DeepSeek, and Grok, respectively.

All models were accessed on the same date (March 10, 2026) using identical zero-shot prompts with no external retrieval. All models were accessed via their official paid web-based interfaces using Google Chrome; retrieval and browsing features were explicitly disabled; temperature and sampling parameters were not modified and are not user-accessible via the standard web interface; prompts were submitted in isolated individual sessions, one topic at a time, with no carry-over context between sessions; outputs were not regenerated regardless of formatting.

This web-interface approach was intentional and chosen to reflect real-world clinical use rather than benchmarking through an application programming interface. Because most clinicians use standard LLM interfaces without adjusting temperature, sampling, or backend routing, this design evaluated the citation accuracy most likely encountered in routine academic practice.

Each model was instructed to answer from training data only and to generate exactly 10 recent (2020 or later) peer-reviewed references per topic in Vancouver citation style, with explicit instruction to avoid fabrication (Fig. 1).

A total of 300 references (10 topics × 3 models × 10 references) were verified against PubMed, DOI resolver (doi.org), Google Scholar, and CrossRef, then independently scored by two NCC-expert raters using a modified Reference Hallucination Scale (12): score 0 = fully accurate; 1 = minor error/citation inaccuracy (typo in author, title, volume, or page); 2 = major error/citation inaccuracy (wrong title, author, journal, or year); 3 = completely fabricated.

Throughout this article, “hallucination” is used as the umbrella term for any citation with a score greater than or equal to 1. Citations containing bibliographic errors were classified as inaccurate, whereas “fabrication” was reserved for references that could not be verified in PubMed, CrossRef, Google Scholar, or via DOI resolution and therefore did not exist as complete bibliographic entities.

We assessed the accuracy of DOI and PMID (pubMed identification number) identifiers for all 300 model-generated references. Each reference was coded for whether the model provided an identifier (DOI_Provided: 1 = yes, 0 = no) and, where an identifier was provided, whether it correctly resolved to a real publication matching the cited title and authorship (1 = correct, 0 = wrong or broken). Identifiers were verified manually via doi.org and PubMed. Identifier provision rates and accuracy rates were compared across models using chi-square tests. To assess calibration, the rate of identifier omission was compared between completely fabricated references (score = 3) and nonfabricated references (score < 3) using Fisher exact test. Identifier status across all four hallucination score categories was examined using a chi-square test for association.

Outputs were anonymized and randomized prior to review to maintain blinding to model identity. Both raters completed a structured calibration exercise before the primary rating phase; discrepancies were resolved by consensus, with the adjudicated score recorded for analysis. Inter-rater reliability was assessed using Cohen kappa (κ). This study involved no human subjects, animal subjects, or identifiable private data and was exempt from institutional review board review.

The primary outcome was the hallucination rate (score ≥ 1, any citation inaccuracy). The secondary outcome was the fabrication rate (score = 3, completely fabricated reference). Hallucination scores were non-normally distributed (Shapiro-Wilk) and reported as medians and interquartile ranges (IQRs); categorical prevalence as frequencies and percentages.

Global inter-model comparison used the Kruskal-Wallis H-test, with Dunn post hoc test (Bonferroni correction, adjusted α = 0.0167). Effect sizes were reported as eta-square (η^2^) and rank-biserial correlation (r). Modified Poisson regression with robust error variances estimated relative risk (RR) and 95% CIs for hallucination (score ≥ 1), high-severity citation inaccuracies (score ≥ 2), and fabrication (score = 3), with DeepSeek as the reference group. To account for the correlation of references generated within the same model-topic session, robust SEs were clustered at the model-topic level (30 clusters of 10 references each). For topic-level analysis, Topic 8 (direct oral anticoagulants [DOAC] reversal in intracerebral hemorrhage [ICH]) served as the hallucination reference (lowest pooled rate) and Topic 10 (blood pressure [BP] management in spontaneous ICH) as the fabrication reference. Inter-model differences within each topic were assessed using Fisher exact test. Topic-specific RRs were visualized using forest plots. All analyses were performed using Python 3.12 (SciPy 1.11, Pandas 2.1, NumPy 1.26). A post hoc power analysis confirmed that primary inter-model comparisons were well powered (> 99% power at α = 0.05), whereas topic-level analyses were underpowered for small-to-moderate differences (estimated power 30–80%), consistent with the wide CIs in the topic-level analyses. All tests were two-tailed; *p* values less than 0.05 was considered statistically significant.

## RESULTS

### Inter-rater Reliability

Agreement between the two raters was excellent (Cohen κ = 0.91; 95% CI, 0.86–0.96; *p* < 0.001), with an observed agreement of 95.3% (286/300 references).

### Overall Hallucination and Fabrication Rates

**Figure 2.**
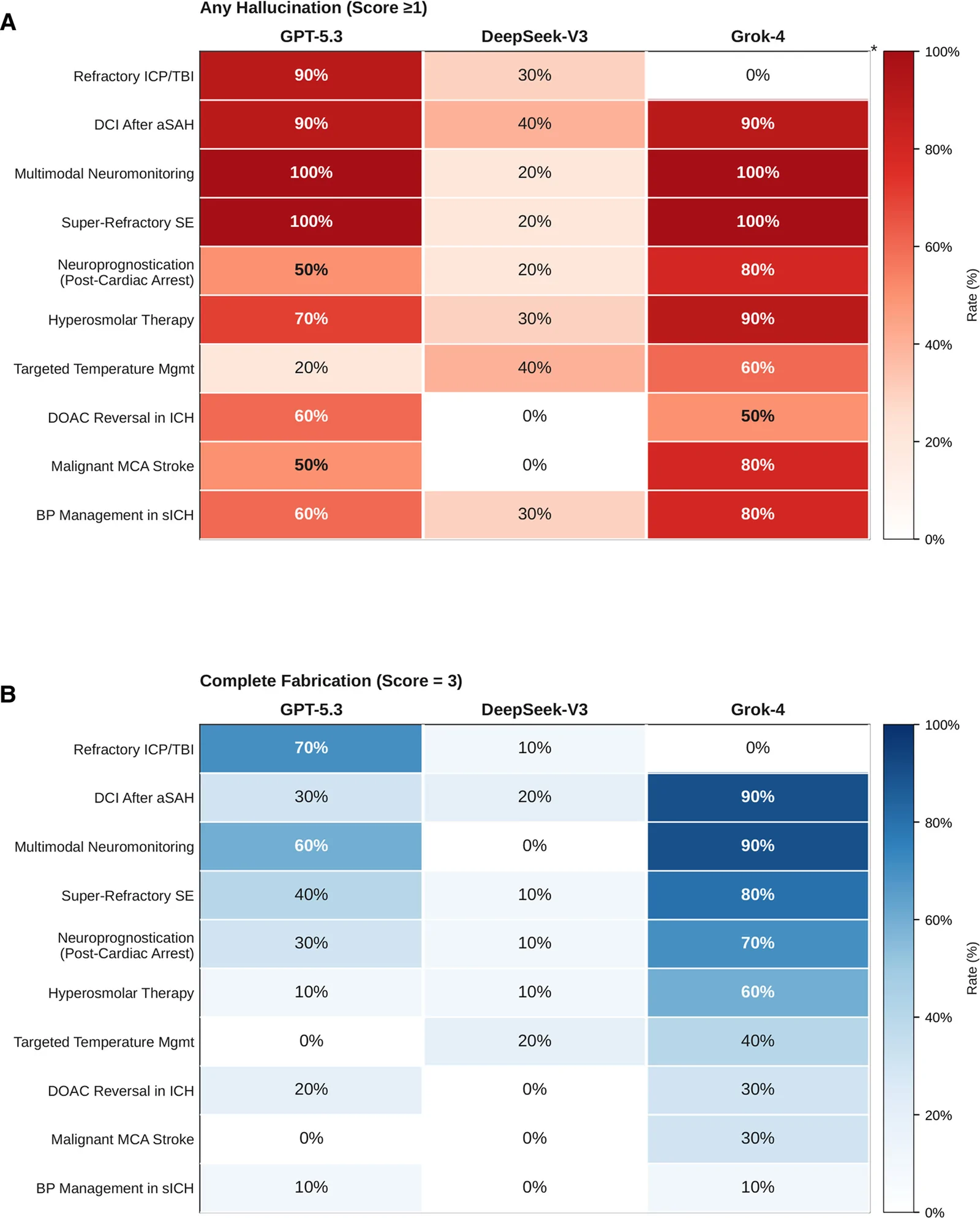
Hallucination and fabrication rates by large language model and neurocritical care topic (*N* = 300). **A**, hallucination rate (score ≥ 1, any citation inaccuracy) per model per topic, expressed as the proportion of 10 references containing any citation inaccuracy. **B**, fabrication rate (score = 3, completely fabricated reference) per model per topic. Color intensity corresponds to citation inaccuracy rate magnitude, *deeper red* indicates higher hallucination rates in (A); *deeper blue* indicates higher fabrication rates in (B). All values represent proportions of 10 references per cell. Generative Pretrained Transformer (GPT) = GPT-5.3 (ChatGPT; OpenAI), DeepSeek = DeepSeek-V3 (DeepSeek AI), Grok = Grok-4 (xAI). aSAH = aneurysmal subarachnoid hemorrhage, BP = blood pressure, DCI = delayed cerebral ischemia, DOAC = direct oral anticoagulant, ICH = intracerebral hemorrhage, MCA = middle cerebral artery, NCC = neurocritical care, SE = status epilepticus, sICH = spontaneous intracerebral hemorrhage, TBI = traumatic brain injury, TTM = targeted temperature management. *For the full description of 10 neurocritical care topics, see Figure 1.

Of 300 references evaluated, 165 (55.0%) contained a verifiable citation inaccuracy (hallucination rate) and 85 (28.3%) corresponded to no existing publication (fabrication rate). Hallucination scores were non-normally distributed across all three models (Shapiro-Wilk, all *p* < 0.001), (**Fig. 2**).

### Model-Level Performance

Median hallucination scores (IQR) were 0.0 (0.0–0.0) for DeepSeek, 1.0 (0.0–3.0) for GPT, and 2.5 (0.0–3.0) for Grok. DeepSeek demonstrated the lowest hallucination rate (23%; fabrication 8%), followed by GPT (69%; fabrication 27%) and Grok (73%; fabrication 50%). High-severity citation inaccuracies (score ≥ 2) occurred in 10% of DeepSeek outputs, 44% of GPT outputs, and 66% of Grok outputs (**Table 1**).

**TABLE 1.** Hallucination (Score ≥ 1) and Fabrication (Score = 3) Rates by Neurocritical Care Topic Across Three Large Language Models (*N* = 300)

Neurocritical Care Topic^a^	GPT-5.3		DeepSeek-V3		Grok-4		*p*
	Median (IQR)^b^	Rate, %	Median (IQR)^b^	Rate, %	Median (IQR)^b^	Rate %	
Any hallucination (score ≥ 1)							
1. Refractory ICP/TBI	3.0 (2.25–3.0)	90	0.0 (0.0–0.75)	30	0.0 (0.0–0.0)	0	< 0.001
2. aSAH DCI	1.0 (1.0–2.75)	90	0.0 (0.0–2.0)	40	3.0 (3.0–3.0)	90	0.014
3. Multimodal Monitoring	3.0 (1.25–3.0)	100	0.0 (0.0–0.0)	20	3.0 (3.0–3.0)	100	< 0.001
4. Super-Refractory SE	2.0 (1.0–3.0)	100	0.0 (0.0–0.0)	20	3.0 (3.0–3.0)	100	< 0.001
5. Neuroprognostication (post-CA)	0.5 (0.0–2.75)	50	0.0 (0.0–0.0)	20	3.0 (2.25–3.0)	80	0.027
6. Hyperosmolar therapy	1.5 (0.25–2.0)	70	0.0 (0.0–0.75)	30	3.0 (1.25–3.0)	90	0.018
7. Targeted temperature management	0.0 (0.0–0.0)	20	0.0 (0.0–1.0)	40	1.5 (0.0–3.0)	60	0.189
8. DOAC reversal in ICH	1.0 (0.0–2.0)	60	0.0 (0.0–0.0)	0	1.0 (0.0–2.75)	50	0.012
9. Malignant MCA stroke	0.5 (0.0–1.0)	50	0.0 (0.0–0.0)	0	2.0 (1.25–2.75)	80	< 0.001
10. BP management in sICH	1.0 (0.0–2.0)	60	0.0 (0.0–0.75)	30	1.5 (1.0–2.0)	80	0.076
Pooled median (IQR) and rate	1.0 (0.0–3.0)	69	0.0 (0.0–0.0)	23	2.5 (0.0–3.0)	73	< 0**.001**
Complete fabrication (Score = 3)							
1. Refractory ICP/TBI	3.0 (2.25–3.0)	70	0.0 (0.0–0.75)	10	0.0 (0.0–0.0)	0	< 0.001
2. aSAH DCI	1.0 (1.0–2.75)	30	0.0 (0.0–2.0)	20	3.0 (3.0–3.0)	90	0.003
3. Multimodal Monitoring	3.0 (1.25–3.0)	60	0.0 (0.0–0.0)	0	3.0 (3.0–3.0)	90	< 0.001
4. Super-Refractory SE	2.0 (1.0–3.0)	40	0.0 (0.0–0.0)	10	3.0 (3.0–3.0)	80	0.007
5. Neuroprognostication (post-CA)	0.5 (0.0–2.75)	30	0.0 (0.0–0.0)	10	3.0 (2.25–3.0)	70	0.018
6. Hyperosmolar therapy	1.5 (0.25–2.0)	10	0.0 (0.0–0.75)	10	3.0 (1.25–3.0)	60	0.014
7. Targeted temperature management	0.0 (0.0–0.0)	0	0.0 (0.0–1.0)	20	1.5 (0.0–3.0)	40	0.082
8. DOAC reversal in ICH	1.0 (0.0–2.0)	20	0.0 (0.0–0.0)	0	1.0 (0.0–2.75)	30	0.186
9. Malignant MCA Stroke	0.5 (0.0–1.0)	0	0.0 (0.0–0.0)	0	2.0 (1.25–2.75)	30	0.036
10. BP Management in sICH	1.0 (0.0–2.0)	10	0.0 (0.0–0.75)	0	1.5 (1.0–2.0)	10	0.585
Pooled median (IQR) and rate	1.0 (0.0–3.0)	27	0.0 (0.0–0.0)	8	2.5 (0.0–3.0)	50	< 0.001

aSAH = aneurysmal subarachnoid hemorrhage; BP = blood pressure; CA = cardiac arrest; DCI = delayed cerebral ischemia; DOAC = direct oral anticoagulant; ICP = intracranial pressure; ICH = intracranial hemorrhage; IQR = interquartile range; MCA = middle cerebral artery; SE = status epilepticus; sICH = spontaneous intracerebral haemorrhage, TBI = traumatic brain injury.

a For full topic descriptions see Figure 1.

b Median (IQR) is derived from the raw citation accuracy scores (0–3) and is identical for any hallucination (score ≥ 1) and complete fabrication (score = 3) sections because both outcomes are binary transformations of the same underlying score distribution.

*p* values (Any hallucination section) from Fisher exact test comparing hallucination rates across the three models per topic. *p* values (Complete fabrication section) from Fisher exact test comparing fabrication rates across the three models per topic. Pooled *p* values from Kruskal-Wallis H-test.

### Global Comparison

The Kruskal-Wallis test confirmed a statistically significant difference in hallucination scores across the three models (H = 68.72, *df* = 2, *p* < 0.001, *η*^2^ = 0.225), representing a large effect size. Post hoc Dunn testing with Bonferroni correction identified significant differences between GPT and DeepSeek (*z* = 5.41, *p* < 0.001, *r* = −0.484) and between Grok and DeepSeek (*z* = 7.55, *p* < 0.001, *r* = −0.576). The difference between GPT and Grok was not statistically significant after correction (*z* = 2.14, *p* = 0.098, *r* = 0.216).

### Identifier Calibration Analysis

The identifier omission increased monotonically with citation severity: 14.1% of accurate references (score = 0) had no identifier vs. 54.1% of fabricated references (score = 3; χ^2^ = 79.07, *p* < 0.001). Fabricated references were significantly more likely to have no identifier (54.1% vs. 26.0%; odds ratio [OR] = 3.35, Fisher exact *p* < 0.001), suggesting partial calibration by GPT-5.3 and Grok-4 but not DeepSeek-V3 (0% omission across its eight fabricated references). Three fabricated references (3.5%) carried a correctly resolving identifier, representing a false reassurance scenario. Full results are presented in **Supplementary Table S1** (https://links.lww.com/CCX/B680).

### Relative Risk

Using DeepSeek as the reference, GPT was 3.00 times more likely to produce any hallucination (95% CI, 1.94–4.63; *p* < 0.001) and Grok was 3.17 times more likely (95% CI, 2.03–4.96; *p* < 0.001) (**Table 2**). For fabrication specifically, GPT carried a 3.38-fold higher risk (95% CI, 1.52–7.49; *p* = 0.003) and Grok a 6.25-fold higher risk (95% CI, 3.07–12.71; *p* < 0.001) compared with DeepSeek. Although Grok-4 had a numerically higher fabrication rate than GPT-5.3 (50% vs. 27%), this difference did not reach statistical significance after accounting for within-cluster correlation (RR 1.85; 95% CI, 0.95–3.59; *p* = 0.069), likely reflecting limited power at the topic-model cell level. For high-severity citation inaccuracies, GPT carried a 4.40-fold higher risk (95% CI, 2.35–8.25; *p* < 0.001) and Grok a 6.60-fold higher risk (95% CI, 3.61–12.08; *p* < 0.001) compared with DeepSeek (**Fig. 2*A*** and ***B***).

**TABLE 2.** Relative Risk of Hallucination (Score ≥ 1) and Fabrication (Score = 3) Among Three Large Language Models

Model Comparison	Any Hallucination (Score ≥ 1)			Complete Fabrication (Score = 3)		
	RR	95% CI	*p*	RR	95% CI	*p*
GPT-5.3 vs. DeepSeek-V3	3.00	1.94–4.63	< 0.001	3.38	1.52–7.49	0.003
Grok-4 vs. DeepSeek-V3	3.17	2.03–4.96	< 0.001	6.25	3.07–12.71	< 0.001
Grok-4 vs. GPT-5.3	1.06	0.76–1.48	0.742	1.85	0.95–3.59	0.069

GPT = Generative Pretrained Transformer, RR = relative risk.

Any hallucination (score ≥ 1). Complete fabrication (score = 3). RRs estimated using modified Poisson regression with robust SEs clustered at the model-topic level (30 clusters of 10 references each) to account for within-session output correlation. RR point estimates are unchanged from the unclustered analysis; CIs are slightly wider, reflecting the clustering adjustment. All *p* values two-tailed. α.05.

### Topic-Level Analysis

**Figure 3.**
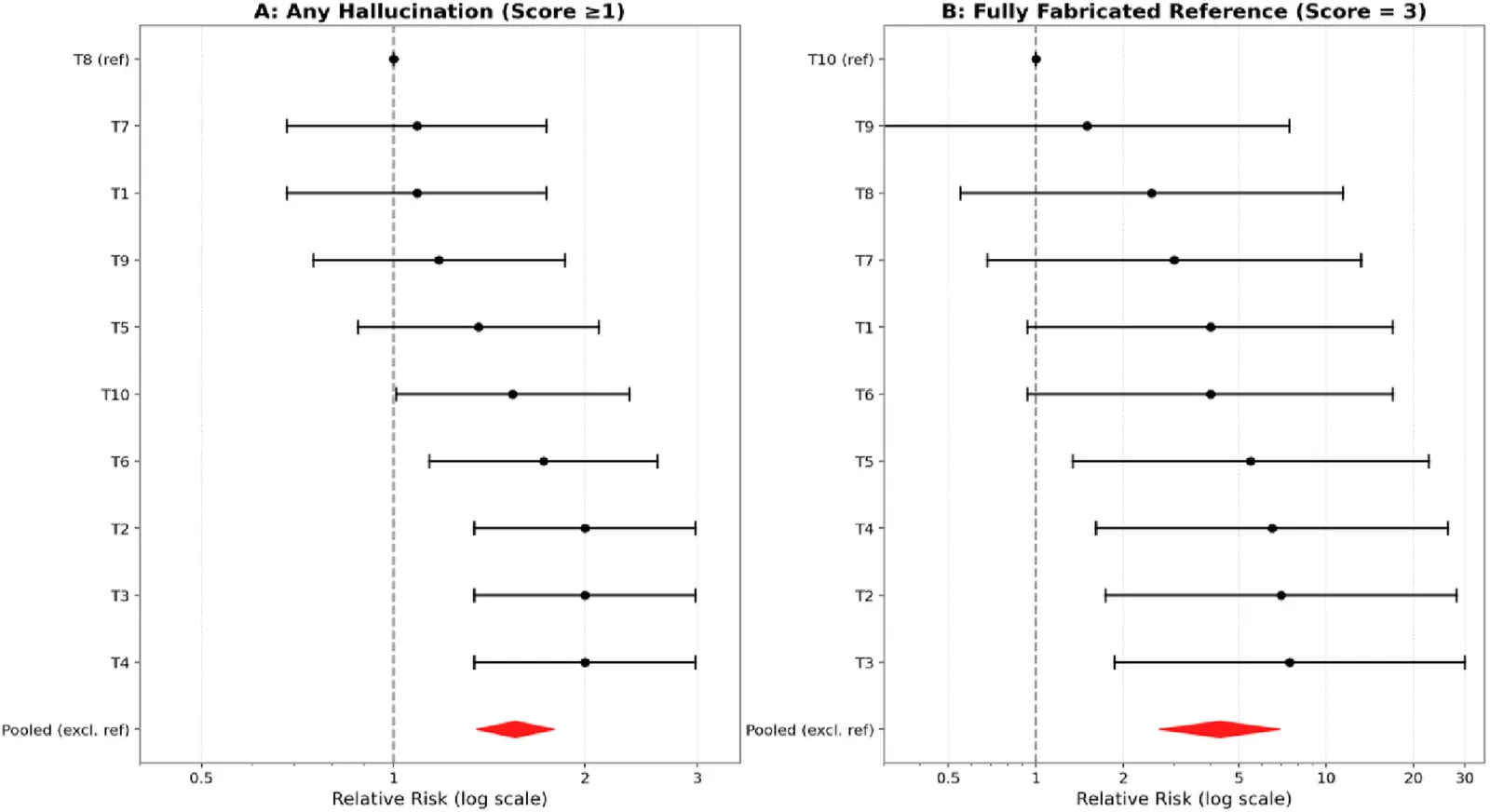
Topic-specific relative risk of hallucination and fabrication across 10 neurocritical care topics. *Forest plots* of topic-specific relative risks of any hallucination (score ≥ 1; **A**) and complete fabrication (score = 3; **B**). A uses direct oral anticoagulants reversal in intracerebral hemorrhage (ICH) (T8) as the reference topic (relative risk = 1.00), selected as the topic with the lowest pooled hallucination rate. B uses blood pressure (BP) management in spontaneous ICH (T10) as the reference topic (relative risk = 1.00), selected as the topic with the lowest pooled fabrication rate. *Horizontal lines* represent 95% CIs. The *red diamond* represents the inverse-variance weighted pooled relative risk across all non-reference topics. Relative risk values are plotted on a logarithmic scale. Relative risks were calculated using modified Poisson regression with robust error variances; *p* values are from Fisher exact test vs. the reference topic. *For the full description of the 10 neurocritical care topics, see Figure 1.

Pooled hallucination rates across all three models ranged from 36.7% (Topic 8, DOAC reversal in ICH) to 73.3% (Topics 2, 3, and 4: cerebral vasospasm/subarachnoid hemorrhage, super refractory status epilepticus [SRSE], and delayed cerebral ischemia). Topics 3 and 4 also had the highest fabrication rates (50.0% and 43.3%, respectively). Topic 10 (BP management in ICH) had the lowest fabrication rate (6.7%). Fisher exact testing identified significant inter-model differences within Topics 1, 3, 4, 8, and 9 (all *p* < 0.05), while Topics 2, 5, 6, 7, and 10 showed no significant pairwise differences after individual topic testing, (**Table 3** and **Fig. 3**). These topic-level estimates are based on 10 references per model-topic cell and should be interpreted with caution given the limited power of individual topic comparisons.

**TABLE 3.** Relative Risk of Hallucination (Score ≥ 1) and Fabrication (Score = 3) by Neurocritical Care Topic

Rank	Neurocritical Care Topic^a^	Pooled Rate, %	Relative Risk	95% CI	*p*
Any hallucination (Score ≥ 1)—ranked highest to lowest RR					
1	2. aSAH DCI	73.3	2.00	1.34–2.98	0.009
1	3. Multimodal monitoring	73.3	2.00	1.34–2.98	0.009
1	4. Super-refractory se	73.3	2.00	1.34–2.98	0.009
4	6. Hyperosmolar therapy	63.3	1.72	1.14–2.60	0.071
5	10. BP management in sICH	56.7	1.54	1.01–2.35	0.196
6	5. Neuroprognostication (post-CA)	50.0	1.36	0.88–2.10	0.434
7	9. Malignant MCA stroke	43.3	1.18	0.75–1.86	0.792
8	7. Targeted temperature management	40.0	1.09	0.68–1.74	0.999
8	1. Refractory ICP/TBI	40.0	1.09	0.68–1.74	0.999
**Ref**	8. DOAC reversal in ICH	36.7	1.00 (ref)	—	—
Complete fabrication (Score = 3)—ranked highest to lowest RR					
1	3. Multimodal monitoring	50.0	7.50	1.87–30.0	< 0.001
2	2. aSAH DCI	46.7	7.00	1.74–28.1	0.001
3	4. Super-refractory se	43.3	6.50	1.61–26.3	0.002
4	5. Neuroprognostication (post-CA)	36.7	5.50	1.34–22.6	0.010
5	1. Refractory ICP/TBI	26.7	4.00	0.94–17.0	0.080
5	6. Hyperosmolar Therapy	26.7	4.00	0.94–17.0	0.080
7	7. Targeted tempeprature management	20.0	3.00	0.68–13.2	0.254
8	8. DOAC reversal in ICH	16.7	2.50	0.55–11.4	0.424
9	9. Malignant MCA Stroke	10.0	1.50	0.30–7.45	1.000
Ref	10. BP management in sICH	6.7	1.00 (ref)	—	—

aSAH = aneurysmal subarachnoid haemorrhage, BP = blood pressure, CA = cardiac arrest, DCI = delayed cerebral ischemia, DOAC = direct oral anticoagulant, ICP = intracranial pressure, ICH = intracranial hemorrhage, MCA = middle cerebral artery, RR = relative risk, Ref = reference category (RR = 1.00), SE = status epilepticus, sICH = spontaneous intracerebral haemorrhage, TBI = traumatic brain injury.

a For full topic descriptions, see Figure 1.

Any hallucination (score ≥ 1), topics ranked from highest to lowest RR. Reference topic: DOAC reversal in ICH (lowest hallucination pooled rate, 36.7%). Complete fabrication (score = 3), topics ranked from highest to lowest RR. Reference topic: BP management in sICH (lowest fabrication pooled rate, 6.7%). Pooled rate (%) = total references meeting the outcome criterion across all three models/30 × 100. RRs calculated using modified Poisson regression with robust SEs. *p* values from Fisher exact test vs. reference topic. All *p* values two-tailed; significance at *p* < 0.05.

## DISCUSSION

Among niche subspecialty clinicians such as NCC who turn to LLMs for point-of-care evidence, more than one-half of all generated citations contained a verifiable citation inaccuracy, and more than one in four did not correspond to any real publication. Many fabricated references were structurally complete, bearing plausible author names, recognizable journal titles, and coherent volume and page designations, and were indistinguishable from legitimate citations, without deliberate cross-database verification.

This distinction matters clinically. A misspelled author name does not alter the evidentiary weight a clinician assigns to a citation. A completely fabricated reference provides no evidentiary basis and cannot be retrieved or verified by any means. In medicine, where decisions are time-sensitive and evidence-based, a clinician who acts on a fabricated meta-analysis or a nonexistent randomized trial is not making an evidence-based decision, regardless of how confidently the information was presented.

The primary clinical concern is not the citation inaccuracy rate in isolation, but its severity profile. Fabricated references, including nonexistent randomized controlled trials (RCTs), nonexistent meta-analyses, and invented guideline updates accounted for 27% of all GPT references and 50% of all Grok references, representing 39% and 68% of their respective hallucinated outputs. Unlike minor citation inaccuracies, these outputs are syntactically and structurally indistinguishable from legitimate literature, carrying realistic author names, plausible journal affiliations, and internally consistent quantitative findings (4, 8, 9).

Our finding of a high hallucination rate across the tested LLMs, particularly for complex medical topics, aligns with previous critical care studies of ChatGPT. A recent study similarly found that ChatGPT recommended extracorporeal membrane oxygenation and deep brain stimulation for elevated intracranial pressure, therapies not indicated for this condition, illustrating how confidently delivered misinformation can lead to harmful decisions (13). These results underscore the warning issued in a recent critical care roadmap: LLM hallucinations and misinterpretations pose tangible patient safety risks, and clinical validation must precede any use of generative AI in ICU settings (14).

Hallucination rates varied substantially across NCC topics, and this variation tracks with the strength and consistency of the underlying evidence base. This finding is potentially multifactorial.

One explanation is that the topics with low hallucination or fabrication rates, target temperature management (TTM) (40% hallucination), DOAC reversal in ICH (36.7% hallucination), and blood pressure management in spontaneous ICH (6.7% fabricated), rest on large, high-quality trials. The TTM2 trial enrolled nearly 1900 patients, ANNEXA-I (Andexanet Alfa in Acute Intracranial Hemorrhage in Patients Receiving an Oral Factor Xa Inhibitor) was the first randomized controlled trial of a DOAC reversal agent for ICH, and BP management in spontaneous ICH is anchored by INTERACT2 and INTERACT3, landmark trials enrolling thousands of patients across multiple continents (15–18).

The high-hallucination topics tell a different story. SRSE is less published, and large prospective trials are lacking; multimodal neuromonitoring has no large RCTs, and a recent consensus noted that a lack of standardization blocks evidence-based development (19, 20). Studies have shown that when training data are limited or conflicting, models may synthesize a fictional middle ground, producing authoritative-sounding statements even when no reliable source exists, not because they intend to deceive, but because they are optimized for coherence rather than accuracy (21). Inconsistent terminology adds another layer of complexity, which could be another reason for more hallucinations in some topics. Terms such as brain tissue oxygen monitoring, Pbto_2_, and cerebral microdialysis are used interchangeably across institutions and time periods, fragmenting the evidence base and confusing any model trying to retrieve citations (22). When the same clinical concept appears under multiple names, an AI model encounters a fragmented and ambiguous signal, even where evidence does exist, a pattern consistent with published observations that inconsistent terminology in specialized domains increases hallucination risk (23, 24). The topic-level pattern observed in this study is consistent with the hypothesis that evidence depth, consistency, and terminology stability may influence citation accuracy; however, this interpretation is exploratory and based on post hoc observation rather than prespecified hypothesis testing.

However, evidence availability is not the only driver. The intrinsic complexity of the clinical query itself also influences hallucination risk. This increases the probability that the model will take a shortcut and fabricate a plausible-seeming citation rather than successfully navigating a complex parametric search (25). Published studies have reported that even state-of-the-art LLMs have failed on differential diagnosis tasks in over 80% of cases, despite achieving high accuracy on final diagnoses, a pattern mirroring our observation that topics with sparse or uncertain evidence produced the highest hallucination rates (26). Our findings contrast with two recent studies that used different methodologies. One study found that OpenAI’s o3 DeepResearch model achieved 83% concordance with meta-analyses and no hallucinations (27), whereas a study of ICU diagnostic accuracy found that DeepSeek was the worst-performing model (28). These key differences likely reflect task design: both contrasting studies used retrieval-augmented generation (RAG) with a live internet search strategy, whereas our zero-shot design asked models to recall citations from training data alone. Unlike standard LLMs that generate answers solely from memory, RAG connects the model to a live, searchable database before responding, like giving a physician access to UpToDate rather than asking them to rely on recall from medical school. Because RAG grounds each answer in retrievable, verifiable documents, it substantially lowers the risk of generating fabricated citations (29).

LLMs generate citations by drawing on patterns learned during training rather than by retrieving verified sources from a live database, as a result, they cannot reliably distinguish a real reference from a plausible-sounding fake (23, 24). While this issue is applicable to any field of medicine, three factors amplify it in more niche subspecialties, such as NCC. First, some NCC topics appear less frequently in the models’ training data. This limited published research, leads to higher citation inaccuracy rates. In contrast, topics with extensive, well-established literature provide a clearer signal and a lower risk of hallucinations. Second, models are often asked to generate citations that fall after their training cutoff date. This temporal mismatch forces them to extrapolate beyond what they actually know, increasing the likelihood of fabrication (23). We acknowledge that temporal mismatch may independently contribute to topic-level variation in hallucination rates and cannot be fully disentangled from evidence density in this cross-sectional design. Third, models are optimized through reinforcement learning from human feedback to produce confident, complete-sounding answers. This training discourages them from expressing uncertainty, even when they lack reliable information. As a result, explicit instructions to avoid fabrication reduce the problem but do not eliminate it (30).

The observed performance gap likely reflects platform- and model-specific differences, but our study cannot determine the mechanism behind this difference. DeepSeek-V3 has been described as a Mixture-of-Experts model pretrained on 14.8 trillion tokens and further optimized through supervised fine-tuning and reinforcement learning post-training stages. These features may partly explain its more consistent citation performance in this study, but this remains uncertain (31).

Its low inter-topic variability (sd = 13.5%, range 0–40%) suggests more consistent performance across the sampled NCC topics, although this finding requires confirmation with repeated sampling and additional topic sets.

Grok’s extreme topic-level variability, including 100% hallucination in NCC Topics 3 and 4 alongside zero rates in NCC Topic 1, may reflect high-variance stochastic output behavior rather than systematic knowledge gaps, though the underlying mechanism cannot be determined from behavioral data alone (24). Although Grok-4’s fabrication rate was numerically higher than GPT-5.3’s (50% vs. 27%), this difference did not reach statistical significance after accounting for within-session output correlation (RR 1.85; 95% CI, 0.95–3.59; *p* = 0.069), and should be interpreted with caution.

A notable behavioral difference was that GPT and Grok consistently omitted DOI and PMID identifiers from their outputs, whereas DeepSeek consistently attempted to report identifiers despite variable accuracy. This pattern is consistent with prior evidence that the DOI field is not performing well in LLM-generated citations across models, while the title remains the most reliably reproduced element (32). Identifier omission by GPT and Grok likely reflects implicit model calibration; these models appear to have learned that omitting an uncertain, arbitrary numeric identifier is preferable to reporting a verifiably incorrect one, a behavior consistent with the known tendency of LLMs to omit high-precision tokens when their training data does not support a confident output (23, 24).

These findings support several evidence-based recommendations for researchers. First, a cross-database verification of every AI-generated citation is suggested, regardless of model selection, as no model achieved an acceptable unverified error rate. Second, RAG architectures should be prioritized over zero-shot parametric recall for literature workflows: by grounding outputs in retrieved, verifiable documents rather than in compressed parametric memory, it directly addresses the corpus sparsity and temporal shift mechanisms documented here, a strategy strongly endorsed in recent prompt engineering guidance for health researchers (33). The limitations of LLMs in high-stakes critical care tasks were recently demonstrated in a real-time evaluation during Society of Critical Care Medicine guideline development, where GPT-4o showed significant gaps when applied to the full clinical evidence synthesis workflow (34). Third, uncertainty-eliciting and chain-of-thought prompting strategies reduce confident fabrication at low implementation cost by prompting the model to check its reasoning before generating an answer, which reduces confident fabrication (35). Fourth, model performance may differ substantially across clinical tasks and workflows. In the present zero-shot citation-generation task, DeepSeek-V3 showed lower hallucination and fabrication rates than the other tested models; however, this behavioral study cannot determine whether this difference reflects architecture, training data, alignment strategy, or interface-level behavior. Recent benchmark studies have likewise emphasized that thorough, ongoing evaluations focused on clinical reasoning capabilities are essential before LLMs can be safely implemented in critical care settings (36).

The identifier calibration analysis partially supports the hypothesis that LLMs signal uncertainty through omission. GPT-5.3 and Grok-4 omitted identifiers for 59.3% and 60.0% of fabricated references vs. 42% and 54% overall (OR = 3.35, *p* < 0.001), suggesting these models were less likely to provide identifiers when fabricating citations, a potential indirect signal of reduced output confidence. DeepSeek-V3 showed no such behavior, omitting identifiers for none of its fabricated references, though its low fabrication rate (8%) limits interpretation. This calibration signal is clinically insufficient: 42.4% of fabricated references still received a wrong identifier, and 3.5% received a correctly resolving one, the most dangerous scenario. Identifier omission alone cannot serve as a safety signal; full-text cross-referencing remains essential.

## LIMITATIONS

This study has several limitations. First, we evaluated a single zero-shot prompt structure without a chain of thought or RAG, representing a conservative baseline that does not reflect optimized deployment scenarios. Second, all models were tested at a single time point, LLMs undergo rapid version updates, and performance may change over time. Third, only 10 NCC topics were selected by a single domain expert without a formal consensus or Delphi process, and although they represent core practice domains, the sample was not stratified by evidence density or guideline availability; the findings may not generalize to all NCC topics, and the observed topic-level hallucination gradient should be interpreted as hypothesis-generating rather than confirmatory. Fourth, LLMs generate responses through a probabilistic process, meaning that repeating an identical prompt does not guarantee an identical output. Exact replication of the specific references generated in this study is therefore not possible. This is a recognized and accepted limitation of all LLM evaluation research and does not invalidate the findings; the hallucination rates and cross-model comparisons reported here reflect a systematic, prespecified sampling of model behavior at a defined time point, consistent with established methodology in this field (37). Although use of commercial web interfaces limits technical reproducibility and prevents control over temperature, hidden system prompts, sampling behavior, or backend routing, this design was intentional and was chosen to reflect the citation accuracy encountered by clinicians using standard LLM interfaces in routine academic practice. Finally, this study primarily evaluated bibliographic accuracy rather than topical relevance, evidentiary quality, or clinical usefulness of cited articles. Although no fully accurate but topically irrelevant citation was identified in this dataset, future studies should incorporate a separate relevance assessment to distinguish citation existence from clinical or topical appropriateness. In addition, each model-topic combination was sampled once; because LLM outputs are stochastic, repeated sampling may provide more stable estimates of model-topic variability. Future studies should also evaluate structured failure typologies for fabricated citations, uncertainty-eliciting and abstention-calibrated prompting strategies, and RAG-enabled comparator arms to estimate residual hallucination rates under current deployment conditions.

In NCC research, LLMs should be used as tools to help summarize and organize information, not as sources of factual knowledge. They are powerful assistants for the communicative dimensions of medical writing, but only under the epistemic oversight of domain-expert clinicians (7, 11).

## CONCLUSIONS

Under standardized zero-shot, retrieval-disabled web-interface conditions, LLMs generated a substantial number of inaccurate and fabricated NCC citations. Error rates differed by model and appeared to differ by topic, although the topic-level findings should be interpreted cautiously. Because fabricated references can look complete and credible, AI-generated citations should be checked across reliable databases before they are used in clinical, educational, or scholarly work. Future work should test retrieval-enabled workflows, repeated sampling, and scoring systems that assess both citation accuracy and topical relevance.

## Supplementary Material
