# Supplementary material for: Hallucination Rate of Peer-Reviewed Citations Generated by Large Language Models in Neurocritical Care
Source: Crit Care Explor. 2026 Aug 25;8(9):e1474. doi: 10.1097/CCE.0000000000001474 (PMC13506236; doi:10.1097/CCE.0000000000001474)
Supplement: Supplementary file 1 [file cc9-8-e1474-s001.pdf]

Supplementary File

Supplementary Table S1. Exploratory Identifier (DOI/PMID) Calibration Analysis (N = 300 references)

Panel A. Identifier accuracy by model. Percentages for "Correct" and "Wrong/Broken" are calculated among references where an identifier was provided. Fabricated reference columns refer to completely fabricated citations (score = 3) only.

Model	N	Identifier Provided n (%)	Identifier Omitted n (%)	Correct /provided n (%)	Wrong/Broken /provided n (%)	Fabricated Refs Correct ID n (%) †	Fabricated Refs Omitted ID n (%) ‡
GPT-5.3	100	58 (58%)	42 (42%)	27/58 (46.6%)	31/58 (53.4%)	2/27 (7.4%)	16/27 (59.3%)
DeepSeek-V3	100	94 (94%)	6 (6%)	53/94 (56.4%)	41/94 (43.6%)	1/8 (12.5%)	0/8 (0%)
Grok-4	100	46 (46%)	54 (54%)	17/46 (37.0%)	29/46 (63.0%)	0/50 (0%)	30/50 (60.0%)
**P value (χ^2)**	—	**$p < .001$**	**$p < .001$**	**$p = .088$**	—	—	—

Identifier provision rates differed across models ($\chi^2 = 55.61$, $p < .001$): DeepSeek-V3 94%, GPT-5.3 58%, Grok-4 46%. Among provided identifiers, accuracy did not differ significantly across models (DeepSeek-V3 56.4%, GPT-5.3 46.6%, Grok-4 37.0%; $\chi^2 = 4.86$, $p = .088$). False reassurance breakdown by model: GPT-5.3 2/27 fabricated refs (7.4%); DeepSeek-V3 1/8 (12.5%); Grok-4 0/50 (0%).

Panel B. Calibration analysis , identifier status stratified by hallucination score. Omission rate increases monotonically with citation severity, suggesting partial calibration by GPT-5.3 and Grok-4.

Score	Definition	N	Identifier Correct n (%)	Identifier Wrong/Broken n (%)	Identifier Omitted n (%)
0	Fully Accurate	135	80 (59.3%)	36 (26.7%)	19 (14.1%)
1	Minor Error	45	8 (17.8%)	20 (44.4%)	17 (37.8%)
2	Major Error	35	6 (17.1%)	9 (25.7%)	20 (57.1%)
3	Fabricated	85	3 (3.5%)	36 (42.4%)	46 (54.1%)
**Total**	**All scores**	**300**	**97 (32.3%)**	**101 (33.7%)**	**102 (34.0%)**
**P value (χ^2)**	ID status vs score category	—	**$p < .001$**	—	—

† False reassurance: a fabricated reference (score = 3) paired with a DOI or PMID that correctly resolves to a real publication, creating the appearance of verifiability for a non-existent citation.

‡ Appropriate abstention: a fabricated reference for which the model provided no identifier, consistent with implicit uncertainty signaling. Fabricated references were significantly more likely to have no identifier than non-fabricated references (54.1% vs 26.0%; OR = 3.35, Fisher's exact $p < .001$).

Identifier correctness verified manually via doi.org and PubMed (accessed April–May 2026). A correct identifier resolves to a real publication matching the cited title and authorship.

Chi-square tests compare provision rates and accuracy rates across models (Panel A) and identifier status across hallucination score categories (Panel B).

DOI = digital object identifier; PMID = PubMed identifier; OR = odds ratio; χ^2 = chi-square; ID = identifier.
